# Ovarian Reserve and Serum Concentration of Thyroid Peroxidase Antibodies in Euthyroid Women With Different Polycystic Ovary Syndrome Phenotypes

**DOI:** 10.3389/fendo.2020.00440

**Published:** 2020-07-28

**Authors:** Agnieszka Adamska, Agnieszka Łebkowska, Anna Krentowska, Justyna Hryniewicka, Marcin Adamski, Monika Leśniewska, Aleksandra Maria Polak, Irina Kowalska

**Affiliations:** ^1^Department of Endocrinology, Diabetology and Internal Medicine, Medical University of Białystok, Białystok, Poland; ^2^Department of Internal Medicine and Metabolic Diseases, Medical University of Białystok, Białystok, Poland; ^3^Faculty of Computer Science, Bialystok University of Technology, Białystok, Poland; ^4^Department of Reproduction and Gynecological Endocrinology, Medical University of Białystok, Białystok, Poland

**Keywords:** PCOS phenotypes, AMH, TPOAbs, fertility, autoimmunity

## Abstract

**Objective:** It has been shown that women with polycystic ovary syndrome (PCOS), as well as Hashimoto's thyroiditis (HT), are characterized by increased incidence of infertility. Serum anti-Müllerian hormone (AMH), which reflects ovarian reserve, is elevated in PCOS women and is decreased in women with HT. The Rotterdam criteria recognize four clinical PCOS phenotypes, i.e., phenotypes A, B, C, and D. The aim of the present study was to investigate the relation between serum concentrations of thyroid peroxidase antibodies (TPOAbs) and ovarian reserve in different PCOS phenotypes.

**Patients and methods:** We examined 141 women with PCOS [phenotype A was diagnosed in 67 (47.5%) women, phenotype B in 30 (21.3%), phenotype C in 28 (19.9%), and phenotype D in 16 (11.3%)] and 88 control subjects of similar age; all women were euthyroid. Serum concentrations of AMH, thyroid-stimulating hormone (TSH), thyroid hormones, and TPOAbs were assessed.

**Results:** We observed positive serum TPOAbs in 21.9% women with PCOS and in 23.9% controls (*p* = 0.07). We did not find differences in the frequency of detection of positive serum TPOAbs between phenotypes A, B, and C and the control group (*p* > 0.05). We did not observe a difference in AMH levels between TPOAbs-positive and TPOAbs-negative women, both in the control group and the PCOS women (all *p* > 0.05). However, serum AMH concentration was markedly higher in the whole PCOS group (*p* < 0.01) and in phenotype A (*p* < 0.01) vs. controls when the serum concentration of TPOAbs was negative. In the groups with positive serum levels of TPOAbs, serum concentration of AMH did not differ between PCOS phenotypes and controls (*p* = 0.23). Additionally, we observed that serum AMH concentration was related to the level of TPOAbs in the PCOS group (*r* = −0.4, *p* = 0.02).

**Conclusions:** The frequency of serum detection of positive TPOAbs did not differ between PCOS phenotypes with clinical/biochemical hyperandrogenism and the control group. The observation of the difference in serum AMH between the PCOS and control groups only in TPOAbs negative women together with the inverse relation of TPOAbs with serum AMH only in the PCOS group might suggest that ovarian reserve is influenced by TPOAbs in PCOS.

## Introduction

Polycystic ovary syndrome (PCOS) is a complex multifactorial disorder characterized by ovulatory dysfunction, hyperandrogenism (clinical and/or biochemical), and characteristic ultrasonography (USG) image of the ovaries (1). According to the Rotterdam criteria, there are four clinical PCOS phenotypes. The most prevalent is phenotype A (2), which is characterized by clinical and/or biochemical hyperandrogenism (HA), menstrual dysfunction (oligo/amenorrhea) (OA), and polycystic ovarian morphology (PCOM) in USG. Phenotype B is recognized when HA+OA are present, phenotype C is characterized by HA+PCOM, and the fourth phenotype, D, is defined by OA+PCOM (3).

An increasing body of evidence suggests the relationship between autoimmunity and PCOS (4–6). Previously, it has been postulated that anti-granulosa cell antibodies might be responsible for the development of PCOS (7). However, this concept has not been confirmed by other researchers (8). Nevertheless, studies emphasize the association between PCOS and autoimmune diseases, especially Hashimoto's thyroiditis (HT) (9). It has been shown that the frequency of autoimmune thyroid disease is 18–40% in PCOS women, depending on the applied diagnostic criteria for PCOS and the patients' ethnicity (5). The etiology of HT is complex and based on abnormal interaction between thyrocytes, T cells, and antigen-presenting cells. HT is characterized by TH1-type mediated autoimmunity and, in consequence, leads to lysis of thyrocytes (10). The trigger factors of abnormal autoimmune response could be non-genetic, e.g., environmental and hormonal factors in genetically predisposed individuals (10). The diagnosis of HT is based on the presence of thyroid peroxidase antibodies (TPOAbs) and/or antibodies against thyroglobulin (TgAbs), hypoechogenic structure of the thyroid in the USG, as well as a variable level of hematopoietic mononuclear cells, especially lymphocytic infiltration (11). HT is the main cause of hypothyroidism in young women, although clinical presentation commonly includes euthyroidism and subclinical hypothyroidism, and ~75% of the patients are euthyroid at the moment of diagnosis (11). Importantly, measurable TPOAbs and/or TgAbs could be present for years without thyroid dysfunction (5). The best serological marker to establish a diagnosis of HT is the serum TPOAbs level. They are present in about 95% of patients with HT and are rare in healthy population. TgAbs are less sensitive and less specific and are detected in about 60–80% patients with HT (11).

Anti-Müllerian hormone (AMH) belongs to the transforming growth factor β family (TGF-β). It is a homodimeric glycoprotein secreted by granulosa cells of pre-antral and early antral ovarian follicles. It is involved in the regulation of follicle growth, inhibiting the recruitment of primordial follicle. It also inhibits aromatase expression, which leads to a decrease in granulosa cell sensitivity to follicle stimulating hormone (FSH) (12). There is a strong correlation between the serum levels of AMH and the number of antral follicles; therefore, the serum AMH level is a biomarker for ovarian reserve and a prognostic marker for fertility (13).

An increased number of pre-antral and antral follicles are observed in women with PCOS, which leads to higher serum AMH concentrations in this group in comparison to women with normal ovaries (14), whereas diminished ovarian reserve has been observed in HT (4). Despite the fact that HT is more frequent in PCOS and women are characterized by increased incidence of infertility, ovarian reserve has not been frequently studied in this group (15). Moreover, there are no data assessing the serum levels of AMH and TPOAbs in different PCOS phenotypes. The present study aimed to investigate the relation between serum concentrations of TPOAbs and ovarian reserve in different PCOS phenotypes.

## Subjects and Methods

### Ethic Approval

All the procedures used in the present study were performed in accordance with the 1964 Helsinki Declaration and its later amendments and other relevant guidelines and regulations. The study was approved by the Ethics Committee of the Medical University of Białystok, Białystok, Poland (approval no. R-I-002/300/2015). The participation in the study was voluntary and free. All the participants signed written informed consents, and the purpose and nature of all the procedures were fully explained prior to the study.

### Subjects

A prospective, cross-sectional study was conducted between January 2016 and May 2019. One hundred forty-one women with PCOS divided into four phenotypes and 88 control subjects of comparable age were included in the study. In the entire group, nine women were treated with L-thyroxine because of HT (six women with PCOS and three women from the control group). We diagnosed PCOS according to the 2003 Rotterdam ESHRE/ASRM PCOS Consensus Workshop Group diagnostic criteria (16). Polycystic ovary syndrome was diagnosed in the presence of at least two of the following criteria: (1) clinical and/or biochemical hyperandrogenism, (2) oligomenorrhea or anovulation, and (3) polycystic ovaries on ultrasound (≥12 follicles measuring 2–9 mm in diameter or ovarian volume >10 ml in at least one ovary). Women with PCOS were recruited from the Department of Endocrinology, Diabetology and Internal Medicine and the Department of Internal Medicine and Metabolic Diseases, Medical University of Białystok, as well as among students. Control subjects were recruited via advertisements. All women were non-smoking. The exclusion criteria were as follows: cardiovascular disease; other causes of menstrual irregularity and/or androgen excess (e.g., hyperprolactinemia, Cushing's syndrome, late-onset congenital adrenal hyperplasia, pregnancy, and breastfeeding); type 1 or type 2 diabetes; chronic or acute infection (present within the previous 30 days), any other serious medical condition; and use of hormonal contraception and/or anti-androgen therapy (within the previous 6 months). Additionally, participants taking medications that could affect glucose or lipid metabolism were excluded from the study.

### Study Protocol

The study protocol was the same for PCOS patients and healthy women. All laboratory studies were performed in the morning, after an overnight fast. In the control group and in spontaneously menstruating PCOS patients, the studies were performed during the early follicular phase (3–5 days) of their menstrual cycles. In amenorrheic PCOS women, the analyses were performed independently of the cycle phase. Clinical examination was performed in all women. Clinical hyperandrogenism was defined as hirsutism (more than eight points in the modified Ferriman–Gallwey score) and/or the presence of acne. Oligo/amenorrhea and anovulation were defined as less than six menses during the previous year. All subjects underwent an oral glucose tolerance test (OGTT) with 75 g of glucose.

### Biochemical Analyses

The concentrations of serum insulin, plasma glucose, total cholesterol (TC), high-density lipoprotein cholesterol (HDL-C), and triglycerides (TG) were measured as previously described (17). Plasma low-density lipoprotein cholesterol (LDL-C) was calculated with Friedewald's formula. Luteinizing hormone (LH), FSH, prolactin (PRL), and sex hormone–binding globulin (SHBG) were assessed as previously described (17). Total testosterone concentration was assessed by radioimmunoassay (DIAsource ImmunoAssays S.A., Belgium); the minimum detectable concentration was 0.05 ng/ml, and the intra-assay and inter-assay coefficients of variation were estimated at 3.3 and 4.8%, respectively. The free androgen index (FAI) was calculated as serum total testosterone (nmol/L) × 100/SHBG (nmol/L) ratio (18). Serum concentration of estradiol was determined by radioimmunoassay (DIAsource ImmunoAssays S.A., Belgium) (minimum detectable concentration−2.7 pg/ml, intra-assay and inter-assay CV−4.7 and 10.4%, respectively). Serum TSH concentration was measured with the immunoradiometric method (sensitivity 0.025 μIU/ml; intra-assay CV−0.6%; inter-assay—CV 2.1%), serum-free T3 (fT3) (sensitivity 0.3 pg/ml; intra-assay CV−6.4%; inter-assay CV−5.5%), and the serum-free T4 (fT4) (sensitivity 0.03 ng/dl; intra-assay CV−10.3%, inter-assay—CV 7.6%); concentrations were detected with radioimmunoassay kits (DIAsource ImmunoAssays S.A., Belgium). Euthyroidism was defined as having normal levels of TSH (reference range, 0.35–4.5 μIU/ml), fT3 (reference range, 2.3–4.2 pg/ml), and fT4 (reference range, 0.89–1.76 ng/dl). TPOAbs concentrations were measured with radioimmunoassay kits (ThermoFisher Scientific, Germany) (sensitivity 5.5 U/ml; intra-assay CV−3.9%; inter-assay—CV 4.1%). TPOAbs were considered as positive if levels were more than 60 U/ml. Serum AMH concentrations were determined by enzyme immunoassay (Beckmann Coulter). The lowest concentration of AMH detectable with a 95% probability was 0.08 ng/ml. The intra-assay and inter-assay CVs were below 5.4 and 5.6%, respectively.

### Calculations

The homeostasis model assessment of insulin resistance (HOMA-IR) was calculated as follows: (fasting insulin (μIU/ml) × fasting plasma glucose (mmol/L))/22.5 (19).

### Ultrasonography of the Thyroid Gland

Ultrasound of the thyroid gland was performed in every patient with the use of a 7.5-MHz linear transducer (Philips HD5 Diagnostic Ultrasound System, Bothell, Washington, USA, Neusoft Park, Hun Nan Industrial Area, Shenyang 110179, China). Thyroid volume (TV) was calculated using the equation: (length × width × thickness of the lobes) × 0.479 (20).

### Ultrasonography of the Ovaries

All women underwent ultrasonographic assessment of the ovaries in the early follicular phase of the menstrual cycle. The procedure was performed by the same gynecologist with a 5–9 MHz transvaginal transducer (Voluson 730 Expert GE Healthcare). Ovarian volume was calculated with the simplified formula for a prolate ellipsoid (21).

### Statistical Analysis

The statistical analyses were performed using the Statistica 13.3 package (Statsoft, Cracow, Poland). The variables were tested for normal distribution using Shapiro–Wilk test. Due to non-normal distribution of the data, all values were expressed as median and interquartile range. Mann–Whitney U test was used to compare the PCOS and control groups. Non-parametric Kruskal–Wallis test with an appropriate *post hoc* test was used to find the differences between the five groups (i.e., the four PCOS phenotypes and the control group). For categorical variables, chi-square/Fisher's exact tests were performed. Spearman test was used for correlation analysis. A *p* < 0.05 was considered statistically significant.

The retrospective power analysis for the difference of serum AMH concentrations between PCOS and controls with positive and negative TPOAbs has obtained the result of power 0.9.

## Results

Table 1 presents the main clinical and biochemical characteristics of the studied groups. Phenotype A was present in 67 (47.5%) PCOS women, phenotype B was diagnosed in 30 (21.3%), phenotype C was observed in 28 (19.9%), and 16 (11.3%) women had phenotype D.

**Table 1:** Clinical and biochemical characteristics of the studied groups.

		**Phenotypes**				
	**Control group (*n* = 88)**	**A (*n* = 67)**	**B (*n* = 30)**	**C (*n* = 28)**	**D (*n* = 16)**	***P***
Age (years)	25 (22.5–27)	24 (22–27)	24 (22–27)	24 (22.5–28)	25.5 (22.5–27)	0.34
BMI (kg/m^2^)	22.0 (20.7–24.1)	24.3 (21.6–28.5)^a^	24.8 (22.4–26.3)^b^	23.0 (21.7–25.9)	22.3 (19.2–24.6)	<0.01
WC (cm)	77 (72–82)	85 (75–94)^a^	101 (99–107)^b^	79 (74–89)	76 (71–85)	0.003
FSH (IU/l)	5.5 (4.4–6.6)	4.7 (3.4–6.1)	4.8 (3.9–6.1)	5.6 (4.8–6.2)	4.8 (3.8–6.2)	0.16
LH (IU/l)	4.0 (2.9–5.1)	4.8 (3.4–6.7)	3.6 (3.0–4.7)	3.9 (3.0–4.9)	4.5 (3.6–5.6)	0.006
TT (ng/ml)	0.5 (0.4–0.6)	0.8 (0.7–1.0)^a^	0.7 (0.6–0.8)^b^	0.8 (0.6–0.9)^c^	0.6 (0.4–0.6)^h^	<0.01
SHBG (nmol/l)	64 (50–89)	38.8 (27–51)^a^^,^^i^	36 (29–53)^b^^,^^j^	52 (36–63)	63.2 (56–80)	<0.01
FAI	2.7 (1.8–3.4)	6.4 (4.9–10.5)^a^^,^^f^	5.6 (4.1–8.5)^b^^,^^g^	4.9 (3.2–7.4)^c^^,^^e^	2.7 (2.0–3.4)^h^	<0.01
Estradiol (pg/ml)	59 (37–75)	60.3 (40–84)	57 (49–75)	56 (46–70)	46 (13–69)	0.86
PRL (ng/ml)	10.4 (7.8–16.9)	14.3 (8.0–21.5)	12.1 (7.9–15.6)	11.6 (8.5–16.9)	13.3 (7.1–27.6)	0.7
TSH (uIU/ml)	1.7 (1.3–2.4)	1.8 (1.3–2.5)	1.8 (1.3–3.3)	1.9 (1.4–2.5)	2.1 (1.6–2.4)	0.91
fT4 (ng/dl)	1.3 (1.2–1.4)	1.3 (1.1–1.4)	1.3 (1.3–1.5)	1.3 (1.2–1.4)	1.3 (1.1–1.4)	0.16
fT3 (pg/ml)	3.2 (2.7–3.7)	3.5 (3.2–3.9)	3.6 (3.3–4.0)	3.5 (3.0–3.9)	3.2 (2.8–3.8)	0.05
Glucose 0′ OGTT (mg/dl)	92 (87–96)	92 (88–98)	95 (90–100)^f^	89 (83–91)	90 (86–93)	0.01
Glucose 120′ OGTT (mg/dl)	91 (75–100)	98 (83–117)^g^	101 (89–109)^f^	85 (76–96)	83 (78–95)	0.002
Insulin 0′ OGTT (uIU/ml)	8.2 (6.9–10.7)	11.7 (8.9–15.2)^a^^,^^g^	10 (6.9–14.9)	8.5 (7.1–10.3)	8.1 (7.2–10.2)	<0.01
Insulin 120′ OGTT (uIU/ml)	29.1 (20.6–44.5)	41.2 (31.6–77)^a^^,^^g^	32.7 (25.3–53.3)	23.8 (15.4–38.5)	29 (18.1–54.3)	<0.01
HOMA-IR	1.8 (1.5–2.5)	2.7 (1.9–3.9)^a^	2.4 (1.5–3.6)	1.8 (1.4–2.3)	1.9 (1.7–2.5)	0.02
Total cholesterol (mg/dl)	172 (153–191)	179 (161–199)	170 (157–195)	169 (153–182)	181 (148–191)	0.36
HDL-cholesterol (mg/dl)	66 (58–79)	65 (51–74)	63 (53–74)	68 (58–75)	72 (54–85)	0.34
LDL-cholesterol (mg/dl)	90 (73–104)	98 (82–113)	91 (76–104)	86 (68–94)	89 (77–109)	0.16
TG (mg/dl)	56 (43-69)	69 (57–111)^a^	76 (52–102)^b^	67 (50–88)	57 (46-65)	<0.01
AMH (ng/ml)	5.3 (3.1–8.8)	9.6 (6.4–13.9)^a^	7.7 (4.9–15.4)	7.3 (4.6–11)	9.0 (7.5–10.6)	<0.01
OV (cm^3^)	11 (7.7–13.6)	15.6^a^^,^^d^ (13.1–22.1)^a^^,^^d^	10.3 (7.6–13.4)^f^	15.1^c^^,^^f^ (11.6–19.8)^c^	16.7 (9–19)	<0.01
TV (ml)	9.6 (8–13.3)	10.3 (8.1–12.8)	12.1 (9.1–13.5)	9.6 (8.6–13)	11.1 (9.4–15.1)	0.7

*Values are expressed as median (interquartile range)*.

a *p < 0.05 phenotype A vs. control*.

b *p < 0.05 phenotype B vs. control*.

c *p < 0.05 phenotype C vs. control*.

d *p < 0.05 phenotype A vs. B*.

e *p < 0.05 phenotype C vs. phenotype D*.

f *p < 0.05 phenotype B vs. phenotype C*.

g *p < 0.05 phenotype A vs. phenotype C*.

h *p < 0.05 phenotype D vs. phenotypes A, B, C*.

i *p < 0.05 phenotype A vs. phenotype D*.

j *p < 0.05 phenotype B vs. phenotype D*.

*BMI, body mass index; WC, waist circumference; TT, total testosterone; TG, triglycerides; OGTT, oral glucose tolerance test; FSH, follicle-stimulating hormone; LH, luteinizing hormone; FAI, free androgen index; SHBG, sex hormone binding globulin; HOMA-IR, homeostasis model assessment of insulin resistance; TSH, thyroid-stimulating hormone; fT4, free T4; fT3, free T3; AMH, anti-Müllerian hormone; OV, ovarian volume; TV, thyroid volume*.

Serum concentrations of total testosterone were significantly higher in phenotypes A, B, and C in comparison to the control group and phenotype D (all *p* < 0.01). Similarly, FAI was higher in phenotypes A, B, and C in comparison to the control group and phenotype D (all *p* < 0.01) (Table 1). We did not notice differences in serum total testosterone concentration and FAI between TPOAbs-positive and TPOAbs-negative women with PCOS (Table 2). However, PCOS groups with positive TPOAbs as well as with negative TPOAbs presented higher serum total testosterone and FAI vs. TPOAbs-positive and TPOAbs-negative control groups (all *p* > 0.05) (Table 2).

**Table 2:** Comparison of clinical, biochemical, and hormonal parameters of PCOS patients and the control group based on the TPOAbs status.

	**Control group**		**PCOS**		***P***
	**TPOAbs negative (*n* = 67)**	**TPOAbs positive (*n* = 21)**	**TPOAbs negative (*n* = 110)**	**TPOAbs positive (*n* = 31)**	
Age (years)	25 (22–27)	27 (24–29)	24 (22–27)	25.5 (23–30)	0.01 *post-hoc* all *p* > 0.05
BMI (kg/m^2^)	21.8 (20.6–23.4)	22.5 (20.9–24.7)	23.4 (21.4–26.3)^a^	25 (22.2–29)^c^	<0.01 *post-hoc* all *p* < 0.01
WC (cm)	77 (73–82)	77.5 (69.5–83)	80 (74–93)	87 (77–97)^c^^,^^d^	<0.01 *post-hoc* all *p* ≤ 0.01
FSH (IU/l)	5.4 (4.4–6.5)	5.5 (4.4–6.7)	4.8 (3.8–6.0)	5.2 (4.2–6.0)	0.15
LH (IU/l)	4.0 (2.9–5.0)	4.0 (3.2–5.0)	3.9 (3.1–5.1)	5.3 (3.5–7.0)^c^	0.03 *post-hoc p* = 0.03
TT (ng/ml)	0.52 (0.4–0.68)	0.53 (0.38–0.63)	0.7 (0.58–0.88)^a^^,^^b^	0.79 (0.64–1.0)^c^^,^^d^	<0.01 *post-hoc* all *p* < 0.01
SHBG (nmol/l)	63 (46–88)	73 (59–91)	47 (30–61)^a^^,^^b^	44 (25–56)^c^^,^^d^	<0.01 *post-hoc* all p < 0.01
FAI	2.8 (2.0–3.7)	2.0 (1.5–3.0)	5.3 (3.2–8.0)^a^^,^^b^	6.4 (4.3–11.2)^c^^,^^d^	<0.01 *post-hoc* all p < 0.01
Estradiol (pg/ml)	53 (28–71)	67 (56–90)	55 (38–74)	61 (47–81)	0.10
PRL (ng/ml)	11.8 (7.9–22.8)	9.1 (5.5–10.3)	13.2 (8.0–22.2)	10.2 (8–17.5)	0.05
TSH (uIU/ml)	1.7 (1.2–2.4)	1.8 (1.5–2.8)	1.9 (1.3–2.5)	1.8 (1.1–3.3)	0.55
fT4 (ng/dl)	1.2 (1.1–1.3)	1.3 (1.2–1.4)	1.3 (1.2–1.4)	1.2 (1.1–1.3)	0.06
fT3 (pg/ml)	3.1 (2.7–3.6)	3.3 (2.4–3.7)	3.5 (3.1–3.8)^a^	3.5 (2.9–3.8)	0.03 *post-hoc* p=0.04
Glucose 0′ OGTT (mg/dl)	91 (87–96)	93 (88–96)	92 (89–98)	89 (85–97)	0.42
Glucose 120′ OGTT (mg/dl)	93 (78–101)	90 (75–99)	95 (83–105)	93 (78–125)	0.43
Insulin 0′ OGTT (uIU/ml)	8.3 (6.8–11.1)	7.7 (7.3–10.2)	10 (7.6–14)	10.2 (7.1–14.4)	0.03 *post-hoc* all *p* > 0.05
Insulin 120′ OGTT (uIU/ml)	31.8 (21.2–47.2)	25.4 (18.8–31.8)	38.2 (24.8–60.7)^b^	30.4 (18.1–62.9)	0.02 *post-hoc p* = 0.04
HOMA-IR	1.8 (1.5–2.5)	1.8 (1.6–2.4)	2.1 (1.6–3.2)	2.3 (1.4–3.7)	0.25
Total cholesterol (mg/dl)	170 (153–191)	180 (148–195)	173 (160–194)	175 (157–198)	0.57
HDL-cholesterol (mg/dl)	65 (58–79)	70 (60–78)	68 (54–77)	59 (50–74)	0.30
LDL-cholesterol (mg/dl)	87 (71–105)	94 (80–100)	91 (78–108)	91 (79–113)	0.43
TG (mg/dl)	58 (42–77)	49 (44–63)	65 (52–92)^a^	68 (52–106)^c^^,^^d^	<0.01 *post-hoc* all *p* < 0.05
AMH (ng/ml)	5.3 (3.4–8.6)	5.1 (2.8–9.8)	8.6 (5.9–12.2)^a^^,^^b^	8.3 (4.8–13.2)^c^	<0.01 *post-hoc* all *p* ≤ 0.04
OV (cm^3^)	11 (7.8–13.7)	10.4 (7.5–12.8)	14.3 (10.6–20.2)^a^^,^^b^	15.5 (10.9–18.6)^c^^,^^d^	<0.01 *post-hoc* all *p* < 0.01
TV (ml)	9.6 (7.9–12.3)	11.4 (8.7–14.9)	10 (8.3–12.8)	10.5 (8.8–13.6)	0.47

*Values are expressed as median (interquartile range)*.

a *p < 0.05 PCOS with negative TPOAbs vs. control with negative TPOAbs*.

b *p < 0.05 PCOS with negative TPOAbs vs. control with positive TPOAbs*.

c *p < 0.05 PCOS with positive TPOAbs vs. control with negative TPOAbs*.

d *p < 0.05 PCOS with positive TPOAbs vs. control with positive TPOAbs*.

*BMI, body mass index; WC, waist circumference; TT, total testosterone; TG, triglycerides; OGTT, oral glucose tolerance test; FSH, follicle-stimulating hormone; LH, luteinizing hormone; FAI, free androgen index; SHBG, sex hormone binding globulin; HOMA-IR, homeostasis model assessment of insulin resistance; TSH, thyroid-stimulating hormone; fT4, free T4; fT3, free T3; AMH, anti-Müllerian hormone; OV, ovarian volume; TV, thyroid volume*.

**Figure 1:**
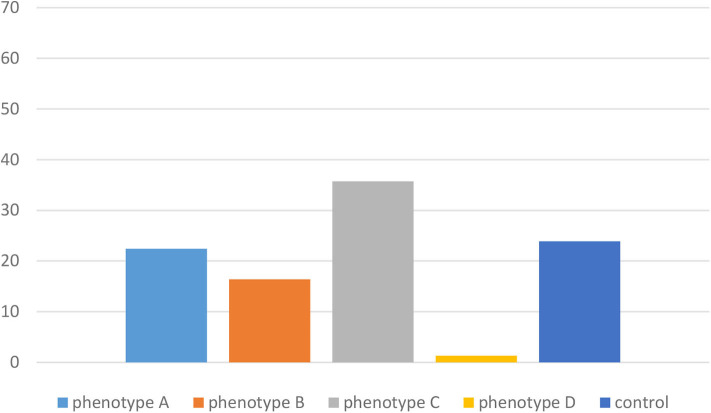
Frequency (%) of positive TPOAbs in different PCOS phenotypes and the control group.

No significant differences were noted between the PCOS phenotypes and the control group in terms of thyroid function tests (TSH, fT3, and fT4) and thyroid volume (all *p* > 0.05) (Table 1). We observed positive serum TPOAbs in 31 (21.9%) women with PCOS and in 21 (23.9%) controls (*p* = 0.07). We did not observe differences in the frequency of detection of positive serum TPOAbs between phenotype A (15 women, 22.4%), phenotype B (5 women, 16.7%), phenotype C (10 women, 35.7%), and the control group (21 women, 23.9%) (*p* > 0.05). Interestingly, only one woman had positive serum TPOAbs in phenotype D (Figure 1). When we divided groups in terms of TPOAbs presence, we did not notice significant differences of serum concentration of TSH, fT4, and thyroid volume in positive and negative TPOAbs PCOS vs. positive and negative TPOAbs control group (*p* > 0.05). However, fT3 was higher in PCOS women with negative TPOAbs vs. the control group with negative TPOAbs (*p* = 0.04) (Table 2). We observed HT structure in thyroid USG in 15% of PCOS women and 19% of the control group (*p* > 0.05).

We noticed higher ovarian volume in phenotypes A (*p* < 0.01) and C (*p* = 0.01) in comparison to the control group, and higher in phenotype A in comparison to phenotype B (*p* < 0.01) as well as in phenotype C vs. phenotype B (*p* = 0.01). When we divided groups in terms of TPOAbs presence, we noticed higher ovarian volume in phenotype A vs. controls and vs. phenotype B (all *p* < 0.01) in the group with negative TPOAbs (Table 1). We did not find differences in ovarian volume between TPOAbs-positive and TPOAbs-negative women with PCOS. However, PCOS groups with positive TPOAbs as well with negative TPOAbs presented higher ovarian volume vs. respective TPOAbs-positive and TPOAbs-negative control groups (all *p* > 0.05) (Table 2).

Serum AMH concentration was markedly higher in the whole PCOS group (*p* < 0.01) and in phenotype A (*p* < 0.01) vs. controls when the serum concentration of TPOAbs was negative. However, in the groups with positive serum levels of TPOAbs, serum concentrations of AMH did not differ between PCOS phenotypes and controls (*p* = 0.23). We found that the serum concentration of AMH correlated with ovarian volume in phenotypes B (*r* = 0.4, *p* = 0.03) and C (*r* = 0.4, *p* = 0.02).

We did not observe relationships between the serum concentration of AMH and TPOAbs separately in phenotypes A, B, and C and in the control group (all *p* > 0.05). However, we observed that serum AMH concentration was related to the level of TPOAbs in the whole PCOS group (*r* = −0.4, *p* = 0.02).

When we divided the whole group into TPOAbs-positive and TPOAbs-negative, higher serum estradiol concentration and lower PRL levels were observed in the TPOAbs-positive group (*p* = 0.02; *p* = 0.02; respectively). Accordingly, we found relationships between TPOAbs levels and estradiol (*r* = 0.20, *p* = 0.001) and PRL (*r* = 0.24, *p* = 0.001) concentrations in the whole studied group.

However, we did not observe correlations between TPOAbs and serum total testosterone concentration or FAI in PCOS phenotypes as well as the control group (all *p* > 0.05).

## Discussion

In the present study, we did not find differences in the frequency of serum detection of positive TPOAbs in women with PCOS and controls. Additionally, differences in frequency of detection of positive serum TPOAbs between phenotype A, phenotype B, and phenotype C as well as the control group were not observed. Interestingly, women presenting phenotype D were characterized by the lowest frequency of occurrence of positive TPOAbs. Conflicting results concerning thyroid-specific antibodies in PCOS patients compared to controls have been demonstrated. In some studies, TPOAbs/TgAbs levels were higher (5, 22–25); in other studies, the authors did not find any differences (26–28). Similarly to our observation, Anaforoglu et al. did not observe any difference in TPOAbs serum levels between the PCOS and control groups (27). However, in a recent meta-analysis, the prevalence of HT was higher in PCOS women [315 (26.03%) out of 1,210 PCOS women] vs. controls [in 96 (9.72%) out of 987 women] (5). Janssen et al. (23) reported elevated TPOAbs or TgAbs in 26.9% of the PCOS patients and in only 8.3% of the controls. Moreover, hypoechoic tissue pattern typical of autoimmune thyroiditis on thyroid USG was present in 42.3% of the PCOS women in that study as opposed to 6.5% of the controls. These observations are similar to our study regarding the positive TPOAbs frequency (21.9%) in PCOS women. However, in our study, we observed an HT pattern in thyroid USG in 16% of PCOS women and in 19% of the control group, whereas in another study, conducted by Singla et al. they found higher serum TPOAbs levels, larger volumes of thyroid gland, as well as more hypoechogenic thyroid in PCOS women vs. controls. However, similarly to our study, TPOAbs have been presented in 27% of PCOS women (24). Accordingly, Garelli et al. observed (25) that HT was present in 27% of women with PCOS in comparison to 8% of the controls. Other findings also show a potential link between PCOS and autoimmunity (7, 29). It has been published that the prevalence of HT depends on race (30), and the higher risk of HT in PCOS women has been noticed in Asians, Europeans, and South Americans (5). As it was mentioned previously, we also observed high positive detection of TPOAbs in the control group (in 23.9% women). Therefore, we can hypothesize that a higher number of TPOAbs-positive women in the control group could be explained by ethnicity, as we enrolled in our study only Caucasian women. Moreover, we included women with and without HT to our study; therefore, it is representative of the population with late-phase HT, whereas in the previous study, one of the exclusion criteria was thyroid disease (27). Additionally, we can explain the higher number of TPOAbs-positive subjects in the control group in comparison to other studies by the fact that most of the cited studies were conducted in earlier years. Currently, an increasing number of people suffer from autoimmune diseases (31), which is why we observe higher frequency of positive TPOAbs in the control group. However, it is hard to explain the lowest frequency of TPOAbs in phenotype D observed in our study. This phenotype is characterized by oligomenorrhea and PCOM in the USG of the ovaries and the lowest level of total testosterone and FAI in comparison to phenotypes A, B, and C. On the contrary, hypothyroidism was found to be less common during follow-up in PCOS patients with persistent hyperandrogenism after menopause, compared with women in general and with patients with Turner's syndrome (32). In another study, the authors concluded that androgens seem to protect against hypothyroidism (33). In our study, we did not observe correlations between serum concentration of TPOAbs and total testosterone or FAI in different PCOS phenotypes. To date, it is not clear if androgens are involved in the prevention of autoimmunity.

In the present study, we observed higher serum AMH concentration in the whole PCOS group, as well as in phenotype A, in comparison to controls when serum concentration of TPOAbs was negative. Interestingly, in the groups with positive serum levels of TPOAbs, the serum concentration of AMH did not differ between PCOS phenotypes and controls. To the best of our knowledge, there is only one study assessing the serum concentration of AMH in TPOAbs-positive and -negative PCOS patients, and the authors did not observe any differences in the compared groups (25). However, they did not divide the PCOS group into phenotypes. Accordingly, in our study, we found that the serum concentrations of AMH and TPOAbs are connected in an inverse manner only in PCOS women. This is a very interesting observation, and the mechanism of this finding is unclear. It has been postulated that human ovaries could be the site of the autoimmune attack of organ-specific as well as organ-nonspecific autoantibodies (8, 34). Monteleone et al. found antithyroid antibodies in ovarian follicular fluid in euthyroid women with HT, which could probably mediate cytotoxicity effect on antral follicles (35). Therefore, we can speculate that the presence of TPOAbs in PCOS women could be responsible for decreasing ovarian reserve by damaging the growing follicles. Additionally, in the study cited above, the authors observed a lower number of oocyte fertilizations in TPOAbs-positive vs. TPOAbs-negative women. In the literature, some reports have supported the theory that there is a potential connection between thyroid autoimmunity and primary ovarian insufficiency (4); however, others did not confirm this hypothesis (36). It has been found that women with PCOS and elevated serum concentration of TPOAbs are at a higher risk of resistance to clomiphene citrate (15), and that in women with unexplained recurrent pregnancy loss, the presence of TPOAbs was a predictor of a reduced live birth rate (37). In contrary, Polyzos et al. reported data from a large cross-sectional study suggesting that HT and hypothyroidism were not associated with reduced ovarian reserve, but rather with low age-specific AMH levels (36). Interestingly, Saglam et al. found higher AMH levels in women with HT treated with LT4 than in controls and lower AMH levels in women with HT without hormone therapy (38). A preventive role of LT4 treatment in women with TPOAbs and euthyreosis in decreasing the miscarriage rate and preterm delivery was also reported (39). Interestingly, Tuten et al. found that the serum concentration of AMH was higher in women with HT in comparison to the control group, but without the difference in terms of antral follicle count between studied groups (40). In contrast to our results, they found a positive relationship between the serum concentration of AMH and TPOAbs. On the contrary, in recently published data, the authors did not find a correlation between the serum levels of AMH and TPOAbs (41). However, they examined adolescent girls without the diagnosis of PCOS, whereas we examined PCOS women with a median age of 25 years. Therefore, autoimmune process probably did not affect ovaries in this young group yet. However, in another retrospective study based on 2,568 women suffering from infertility, it has been shown that the serum concentration of TPOAbs was not associated with ovarian reserve (42). These conflicting results could be connected with the heterogeneity of the groups and with the fact that it was a retrospective study. On the other hand, in the longitudinal population study with 12 years of follow-up, it was observed that women with lower serum concentration of AMH had higher serum levels of TPOAbs at baseline with a tendency to increasing autoimmunity in comparison to women with better ovarian reserve status (43). As it was mentioned in the *Introduction* section, the serum concentration of AMH is considered as one of the most sensitive indicators of ovarian reserve; therefore, we can speculate based on the previous observation that the presence of TPOAbs in women with PCOS observed in our study could be connected with decreased fertility.

The mechanisms driving the autoimmune attack on the thyroid gland are complex, including predominantly genetic, gender-associated, and environmental factors such as iodine supply, medications, chemicals, and infections (5). Extensive data have suggested hormonal influence on the pathogenesis of autoimmune diseases (44). Sex steroids are the most important factor, which may explain higher prevalence of HT in women than in men. Abnormalities in the concentrations of circulating sex hormones are also a feature associated with PCOS. There is a hypothesis that a normal to high serum level of estrogen coupled with low serum concentration of progesterone may be responsible for the development of autoimmunity in PCOS women (44). Additionally, PCOS women often present anovulatory cycles with higher estrogen-to-progesterone ratio, which may have an impact on autoimmune disorders. It could be connected with the stimulatory effect of estrogens on the immune system (45, 46). It has been shown that estrogens increase interleukin-4 expression in TH2 cells, interleukin-1 in monocytes, interleukin-6 in T-cells, and interferon gamma in TH1 cells. In contrast, progesterone exerts inhibitory effects on the immune system by decreasing the proliferation of macrophages and reducing the secretion of interleukin-6 from monocytes (45–47). However, in our study, we did not measure the serum progesterone level because the studies were undertaken in the early follicular phase (3–5 days). Interestingly, we observed higher serum estradiol concentration and lower PRL levels in the TPOAbs-positive group vs. TPOAbs-negative women. We observed a positive relationship between TPOAbs levels and serum concentrations of estradiol, and a negative relationship with the serum levels of PRL in the whole studied group. Aeduc et al. found that the serum levels of estradiol were higher in TPOAbs-positive PCOS women than in TPOAbs-negative ones (22). Therefore, the imbalance between the serum concentrations of estrogens and progesterone may be responsible for HT in women with PCOS (22), and maintaining regular menstrual cycles could reduce the risk of the development of HT in PCOS women. As it was mentioned earlier, we did not observe any differences between phenotypes A, B, and C and controls, and we can speculate that it could be connected with the previous proper treatment of PCOS women with restoring the estrogen–progesterone balance. Therefore, screening and treatment for thyroid disease at the time of PCOS diagnosis and during follow-up are important.

The main limitation of the present study is a relatively small size of the groups representing each phenotype, especially phenotype D. However, this is the first study evaluating ovarian reserve and TPOAbs in different PCOS phenotypes. Additionally, our study was performed in women within euthyroid range to exclude the effects of hypothyroidism. Another possible limitation could be connected with the measurement of total testosterone with the radioimmunoassay method and not with the liquid chromatography–tandem mass spectrometry method. However, liquid chromatography–tandem mass spectrometry is not widely used due to the costs of the measurements.

## Conclusions

On the basis of the obtained results, we concluded that the frequency of serum detection of positive TPOAbs did not differ between PCOS phenotypes with clinical/biochemical hyperandrogenism and the control group. The observation of the difference in serum AMH between the PCOS and control groups only in TPOAbs-negative women together with the inverse relation of TPOAbs with serum AMH only in the PCOS group might suggest that ovarian reserve is influenced by TPOAbs in PCOS. Therefore, when ovarian reserve is needed to be assessed, thyroid autoimmunity should be evaluated at the same time.

## Data Availability Statement

The raw data supporting the conclusions of this article will be made available by the authors, without undue reservation.

## Ethics Statement

The studies involving human participants were reviewed and approved by Ethic Committee of Medical University of Białystok, Białystok, Poland, Approval No. R-I-002/300/2015. The patients/participants provided their written informed consent to participate in this study.

## Author Contributions

AA: the conception and design of the study, acquisition of data, analysis and interpretation of data, and writing the article. AŁ, AK, AP, ML, and JH: acquisition of data. MA: analysis and interpretation of data. IK: analysis and interpretation of data, revising the article, and final approval of the version to be submitted. All authors contributed to the article and approved the submitted version.

## Conflict of Interest

The authors declare that the research was conducted in the absence of any commercial or financial relationships that could be construed as a potential conflict of interest.
